# Effect of a single bout of morning or afternoon exercise on glucose fluctuation in young healthy men

**DOI:** 10.14814/phy2.14784

**Published:** 2021-04-27

**Authors:** Yoshiaki Tanaka, Hitomi Ogata, Insung Park, Akira Ando, Asuka Ishihara, Momoko Kayaba, Katsuhiko Yajima, Chihiro Suzuki, Akihiro Araki, Haruka Osumi, Simeng Zhang, Jaehoon Seol, Keigo Takahashi, Yoshiharu Nabekura, Makoto Satoh, Kumpei Tokuyama

**Affiliations:** ^1^ Graduate School of Comprehensive Human Science University of Tsukuba Tsukuba Ibaraki Japan; ^2^ Graduate School of Humanities and Social Sciences Hiroshima University Hiroshima Japan; ^3^ International Institute for Integrative Sleep Medicine (WPI‐IIIS) University of Tsukuba Tsukuba Ibaraki Japan; ^4^ Department of Somnology Tokyo Medical University Shinjuku Tokyo Japan; ^5^ Department of Nutritional Physiology Faculty of Pharmaceutical Sciences Josai University Sakado Saitama Japan; ^6^ Faculty of Health Science Tsukuba International University Tsuchiura Ibaraki Japan; ^7^ R&D Center for Tailor‐Made QOL University of Tsukuba Tsukuba Ibaraki Japan

**Keywords:** continuous glucose monitoring, exercise timing, glucose fluctuation

## Abstract

The timing of exercise plays an important role in the effect of the exercise on physiological functions, such as substrate oxidation and circadian rhythm. Exercise exerts different effects on the glycemic response to exercise and meal intake depending on when the exercise performed. Here, we comprehensively investigated the effects of the timing (morning or afternoon) of exercise on glucose fluctuation on the basis of several indices: glycemic variability over 24 h (24‐h SD), J‐index, mean amplitude of glucose excursions (MAGE), continuous overall net glycemic action (CONGA), and detrended fluctuation analysis (DFA). Eleven young men participated in 3 trials in a repeated measures design in which they performed a single bout of exercise at 60% of their maximal oxygen uptake for 1 h beginning either at 7:00 (morning exercise), 16:00 (afternoon exercise), or no exercise (control). Glucose levels were measured using a continuous glucose monitoring system (CGMs). Glucose fluctuation was slightly less stable when exercise was performed in the afternoon than in the morning, indicated by higher CONGA at 2 h and α_2_ in DFA in the afternoon exercise trial than in the control trial. Additionally, decreased stability in glucose fluctuation in the afternoon exercise trial was supported by the descending values of the other glucose fluctuation indices in order from the afternoon exercise, morning exercise, and control trials. Meal tolerance following exercise was decreased after both exercise trials. Glucose levels during exercise were decreased only in the afternoon exercise trial, resulting in less stable glucose fluctuations over 24 h.

## Graphical abstract

Comprehensive analyses of multiple indices revealed that the effects of exercise on glucose fluctuation depends on the timing of the exercise. Glucose fluctuation was slightly less stable when exercise was performed in afternoon with higher insulin levels (red) than in morning with lower insulin levels (blue). The effect of differences in the timing of exercise on glucose fluctuation may be due to differences in glucose level changes during exercise.

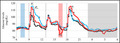

## 1 INTRODUCTION

The timing of exercise critically affects energy metabolism, clock gene expression, and muscle protein synthesis (Aoyama & Shibata, 2017, 2020; Aqeel et al., 2020; Haxhi et al., 2013). Fat oxidation over 24 h is increased by exercise performed before breakfast, but not after breakfast and the subsequent period (Iwayama et al., 2017; Iwayama, Kawabuchi, et al., 2015; Iwayama, Kurihara, et al., 2015; Shimada et al., 2013). Peak timing of the expression of *Bmal1*, a clock gene in the core circadian loop, tends to be advanced by exercise performed in the morning, and tends to be delayed by exercise performed in the afternoon (Tanaka et al., 2020). Skeletal muscle cross‐sectional area is increased by exercise performed in the evening compared with that in the morning (Küüsmaa et al., 2016). Exercise prescription guidelines for patients with diabetes (Colberg et al., 2010) and healthy adults (Garber et al., 2011) have defined the modality, intensity, duration, and frequency of exercise, but have not defined the timing of the exercise. Elucidating the optimal timing of exercise for each purpose would be beneficial toward increasing the benefits achieved by the same amount of exercise, especially for patients who are unable to increase their exercise intensity or duration due to certain restrictions.

The effect of the timing of exercise around meals on postprandial hyperglycemia have been widely discussed. Two reviews discussed a beneficial effect of exercise performed post‐meal on impaired postprandial hyperglycemia (Aqeel et al., 2020; Haxhi et al., 2013), but inconsistent results were also obtained. For example, in previous studies comparing the effects of exercise performed pre‐ and post‐meal in subjects with diabetes, postprandial hyperglycemia was suppressed by post‐meal exercise alone (Colberg et al., 2009; Ruegemer et al., 1990), or pre‐meal exercise alone (Terada et al., 2016), or both pre‐ and post‐meal exercise (Heden et al., 2015). On the other hand, in healthy young men (Hatamoto et al., 2017) and obese men (Farah & Gill, 2013), neither pre‐ nor post‐meal exercise affected postprandial hyperglycemia. These previous studies had 2 types of methodological limitations. In 4 of the studies, the blood glucose sampling frequency was limited to 8 times over 3 h (Colberg et al., 2009), 12 times over 3 h (Ruegemer et al., 1990), 19 times over 4 h (Heden et al., 2015), and 12 times over 7 h (Farah & Gill, 2013). The time course of blood glucose level changes can be more accurately measured using continuous glucose monitors (CGMs), which have been available for almost 2 decades (Rebrin & Steil, 2000). Two studies using CGMs examined various indices, including the 24‐h mean blood glucose level, fasting glucose, and postprandial glucose area under the curve (AUC) (Hatamoto et al., 2017; Terada et al., 2016), but the advantages of this approach were not fully utilized. Several indices are proposed for monitoring glucose fluctuations: glucose variability over 24 h (24‐h SD), J‐index (Wójcicki, 1995), mean amplitude of glucose excursions (MAGE) (Service et al., 1970), and continuous overall net glycemic action (CONGA*n*, for the indicated *n* hours) (McDonnell et al., 2005). These indices were not comprehensively evaluated in previous studies assessing the effects of exercise on glucose fluctuations (Hatamoto et al., 2017; Terada et al., 2016). Furthermore, the effects of exercise have not been investigated by detrended fluctuation analysis (DFA) (Ogata et al., 2006), an extended random walk analysis that characterizes glucose fluctuations.

Glucose variability during exercise may affect the indices of glucose fluctuation. Compared with glucose levels immediately before a single bout of exercise, glucose levels immediately after exercise are not changed by exercise performed in a fasting condition, but are decreased by exercise performed in a fed condition (Costill et al., 1977; Foster et al., 1979; Gaudet‐Savard et al., 2007; Poirier et al., 2001). Previous studies that assessed glucose level changes during exercise, however, did not measure glucose fluctuations over 24 h. Both hyperglycemia (Quagliaro et al., 2003; Wautier et al., 2001) and hypoglycemia (Kajihara et al., 2017; Razavi Nematollahi et al., 2009) induce endothelial dysfunction, which predicts future cardiovascular events (van Sloten et al., 2014). Therefore, glycemic homeostasis must be maintained for the prevention/treatment of endothelial dysfunction and cardiovascular disease induced by endothelial dysfunction.

In the present study, we comprehensively investigated the effects of the timing of exercise (morning or afternoon) on glucose fluctuation in young healthy men and energy metabolism as a secondary outcome.

## 2 MATERIAL AND METHODS

### 2.1 Subjects

Eleven healthy young men participated in the present study after providing informed consent. The subjects had a history of performing moderate exercise fewer than 3 days per week and were not trained individuals. The subjects did not have a medical condition precluding them from the study, were not taking any medications, and did not have a smoking habit at the time of the study. The present study was approved by the ethics committee of the University of Tsukuba (Ref No., Tai 28–32) and registered with Clinical Trials UMIN (ID No. UMIN 000038252).

Leukocyte clock gene expression and core body temperature findings from this experiment were published in another paper (Tanaka et al., 2020).

### 2.2 Pre‐experimental evaluation

To determine the workload corresponding to 60% of the individual maximal oxygen uptake ($\dot{V}$ O_2_max), the subjects performed a graded exercise test comprising submaximal and maximal tests using a cycle ergometer (Aero bike 75XLII; Combi, Tokyo, Japan) (Shimada et al., 2013). The graded exercise test was performed at least 1 week before the first experiment.

### 2.3 Experimental design

The present study was a randomized, repeated measure design comprised of three 24‐h calorimetry trials with a single 1‐h bout of exercise beginning either at 7:00 (morning), 16:00 (afternoon), or without an exercise session (control). The 3 trials were separated by at least 1 week and completed within 2 months. The subjects were asked to maintain their body weight throughout the 3 trials.

Starting 4 days before the beginning of each trial to the end of the trial, the subjects maintained a regular life schedule: wake (6:00), breakfast (9:00), lunch (13:00), dinner (18:00), and sleep (23:00). The schedule was confirmed by continuous activity assessment with a wrist‐worn actigraph (GT3X‐BT, AMI, VA) and a self‐reported log. The day before beginning each trial, a CGMs (iPro2, Medtronic MiniMed, CA) was attached to each subject. At 22:00 on day 1, subjects entered the metabolic chamber and slept for 7 h from 23:00 to 6:00. On day 2, experimental meals were provided at 9:00 as breakfast, 13:00 as lunch, and 18:00 as dinner. Exercise was performed at 60% of $\dot{V}$ O_2_max for 1 h using a cycle ergometer beginning at 7:00 (morning exercise trial), at 16:00 (afternoon exercise trial), or the subjects remained in a sedentary position (control trial). Subjects were asked to remain awake and maintain a sedentary position except during the specified exercise session and sleep period. Subjects stayed in the metabolic chamber until 7:00 on day 3.

Experimental meals were designed to achieve an individual energy balance over 24 h during indirect calorimetry, assuming a resting metabolic rate of 24.0 kcal/kg/day, according to estimated energy requirements for Japanese young men (Ministry of Health, Labour, & Welfare of Japan, 2015). The physical activity factor was assumed to be 1.75 on day 1, 1.60 in the trials with exercise sessions, and 1.25 in the control trials on day 2. The experimental meals comprised 15% protein, 25% fat, and 60% carbohydrate, expressed as a percentage of the total energy intake. The contributions of breakfast, lunch, and dinner to the total 24‐h intake were 33.3%, 33.3%, and 33.4%, respectively.

### 2.4 Glucose monitoring

Glucose levels were continuously measured using a CGMs connected to a glucose sensor (Enlite Glucose Sensor, Medtronic MiniMed, CA). This device measures glucose with a relative mean absolute difference of 11% (Rodbard, 2016). On the day before beginning each trial, the glucose sensor was inserted into the subcutaneous tissue of the abdomen of each subject, and the interstitial glucose level was measured every 5 min until the end of the trial. The recorded interstitial glucose level was converted to blood glucose level by calibrating against the blood glucose level measured from the fingertip 4 times per day (before breakfast, lunch, dinner, and sleep).

To investigate glucose fluctuation, 24‐h SD, J‐index, MAGE, CONGA1, CONGA2, and CONGA4 were calculated from the individual time course of glucose over 24 h in each trial. The 24‐h SD indicates the amount of variation or dispersion of individual data in each trial. The J‐index indicates both the mean and variability of the glucose level and was calculated on the basis of the mean and SD of glucose levels over 24 h as 0.001 (mean + SD)^2^ (Wójcicki, 1995). MAGE was calculated as a mean increase and decrease greater than the 24‐h SD (Service et al., 1970). CONGA*n* was calculated as the SD of all differences in the glucose level for *n* h (McDonnell et al., 2005). For example, CONGA1 was calculated as the SD from the differences in glucose levels as follows: the glucose level at 7:00 minus that at 6:00, glucose level at 7:05 minus that at 6:05, etc., ending with the glucose level at 6:00 minus that at 5:00 the next morning.

The correlation property of the glucose fluctuation was estimated using DFA, which was previously described in detail as an extended random walk analysis (Ogata et al., 2006, 2012, 2013, 2019; Peng et al., 1995). The advantage of DFA is that it can more accurately quantify the correlation properties of the original signals, even if those signals are masked by non‐stationarity compared with traditional methods, such as standard autocorrelation and/or power spectrum analysis (Taqqu et al., 1995). The time course of glucose levels measured by CGMs was prepared with equally sized sliding (1 point at a time) windows of length *n*. For each window, a third‐order polynomial trend, representing non‐stationarity in that window, was fit to the data. Then, the detrended fluctuations *F*(*n*) were calculated as the root mean square deviation from the trend in each window, which was summed for all of the windows of the entire time course analyzed. This procedure was repeated for different window sizes. Finally, the short‐range scaling exponent (α_1_), the long‐range scaling exponent (α_2_), the mean glucose fluctuation (averaged log 10 *Fm*), and the crossover point (window size between α_1_ and α_2_) were defined. White noise (uncorrelated time series) and Brownian motion (integrated white noise) are characterized by α = 0.5 and 1.5, respectively. Similar to the relation between white noise and Brownian motion, the value of α after integration is increased by 1. Therefore, a negatively correlated long‐range fluctuation of increments is represented by 1.0 < α < 1.5 for a measured glucose time series, whereas a positively correlated increment is represented by 1.5 < α < 2.0 (furthermore, α > 2.0 can be explained by repeated integration).

To investigate the glucose response during exercise, the glucose level (mean, minimum, and change during the exercise period [from 7:00 to 8:00 and from 16:00 to 17:00]) were calculated. The change in the glucose level during the exercise period was calculated by subtracting the glucose level immediately before exercise (i.e., 6:55 or 15:55) from either the peak or trough glucose level during the period. To investigate the postprandial glucose responses, MIME ∆*G* and ∆*T* (Service & Nelson, 1980) were calculated from the individual time course of glucose levels in each trial. The MIME ∆*G* and ∆*T* indicate the amount of the increase and the time required to reach the peak glucose level due to meals, respectively.

### 2.5 Plasma insulin level

Blood samples were collected into EDTA‐coated test tubes at 6:00, 9:00 (before breakfast), 12:00, 15:00, 18:00 (before dinner), 21:00, and 23:00 (before sleep) on day 2, and 6:00 on day 3 using an indwelling intravenous catheter. Immediately after collection, the blood samples were centrifuged at 15°C for 15 min, and the plasma samples separated from the whole blood were stored at −55°C until the insulin levels were quantified. Plasma insulin levels were measured by enzyme‐linked immunosorbent assay (Insulin ELISA, ALPCO, Salem, NH). The homeostatic model assessment for insulin resistance (HOMA‐IR) was calculated on the basis of the glucose level measured by the CGM and plasma insulin level at 6:00 on day 2 as glucose (mg/dl) × insulin (mIU/L)/405.

### 2.6 Energy metabolism

Energy metabolism was measured in an airtight chamber (Fuji Medical Science, Chiba, Japan), which measured 2.00 × 3.45 × 2.10 m, with an internal volume of 14.49 m^3^. The chamber was furnished with a small window for blood sampling, bed, desk, chair, toilet, and cycle ergometer. The temperature and relative humidity of the incoming fresh air were controlled at 25.0 ± 0.5°C and 55.0 ± 3.0%, respectively. The oxygen (O_2_) and carbon dioxide (CO_2_) concentrations in the outgoing air from the metabolic chamber were measured using an online process mass spectrometer (VG Prima δB; Thermo Electron, Winsford, UK). Every 5 min, O_2_ consumption ($\dot{V}$ O_2_) and CO_2_ production ($\dot{V}$ CO_2_) rates were calculated using an algorithm providing an improved transient response (Tokuyama et al., 2009). Urine samples were collected for 24 h from 6:00 on day 2 to 6:00 on day 3, and urinary nitrogen excretion (N) was measured using the Kjeldahl method. Energy expenditure and macronutrient oxidation were calculated from the $\dot{V}$ O_2_, $\dot{V}$ CO_2_, and N values (Ferrannini, 1988) during the exercise bouts and for the total over 24 h. Energy balance was calculated by subtracting the total energy expenditure from the total energy intake for 24 h from 6:00 on day 2 to 6:00 on day 3.

### 2.7 Statistics

According to the results of a pilot study, which measured α_2_ in DFA, a sample size calculation using G‐Power 3.1.9 software revealed that 10 subjects would be required to observe a significant difference in glucose fluctuation using 1‐way analysis of variance (ANOVA) with 90% power and a 5% alpha level. To allow for measurement errors or subject dropout, 11 subjects were recruited. The data are shown as means ± SD. Time courses averaged every 15 min of glucose level, energy expenditure, carbohydrate, and fat oxidation were compared by linear mixed‐models ANOVA with post hoc Bonferroni analysis. The results of the subjects’ characteristics, glucose fluctuations (24 h SD, J‐index, MAGE, CONGA*n*), DFA (α_1_, α_2_, *Fm*, and cross over point), glucose response to exercise (mean, minimum, and change in glucose during exercise periods), postprandial glucose responses (MIME∆*G* and ∆*T*), and energy metabolism (energy expenditure and macronutrient oxidation over 24 h) were compared by one‐way ANOVA with post hoc Bonferroni analysis. The difference in energy metabolism during exercise between the morning and afternoon exercise trials was compared by a paired t test. Plasma insulin levels were compared by linear mixed‐models ANOVA with post hoc Bonferroni analysis. Relationships between the changes in the glucose levels during exercise and the pre‐, post‐exercise insulin level, or the pre‐exercise glucose level were determined by Pearson's correlation. All statistical analyses were performed using SPSS statistical software (Version 25; IBM Japan, Tokyo, Japan). The level of significance was set at *p* < 0.05.

## 3 RESULTS

### 3.1 Subjects’ characteristics

The subjects’ characteristics are shown in Table 1. The ages ranged from 22 to 30 years. The HOMA‐IR ranged from 0.53 to 1.77. The HOMA‐IR cutoff point is 2.5, and values above and below 2.5 indicate populations with and without insulin resistance, respectively (Andrade et al., 2016). Body weight, body fat, and HOMA‐IR did not differ significantly among the 3 trials.

**TABLE 1:** Subject characteristics

	Control	Morning exercise	Afternoon exercise
Age	24.5 ± 2.8		
Height (cm)	173.4 ± 6.4		
$\dot{V}$ O_2_max (ml/kg/min)	46.2 ± 7.4		
Body weight (kg)	67.3 ± 7.3	67.3 ± 7.6	67.4 ± 7.4
Body fat (%)	15.6 ± 4.1	16.0 ± 4.2	15.9 ± 3.9
HOMA‐IR	1.10 ± 0.33	1.12 ± 0.41	1.02 ± 0.23

### 3.2 Glucose levels

**FIGURE 1:**
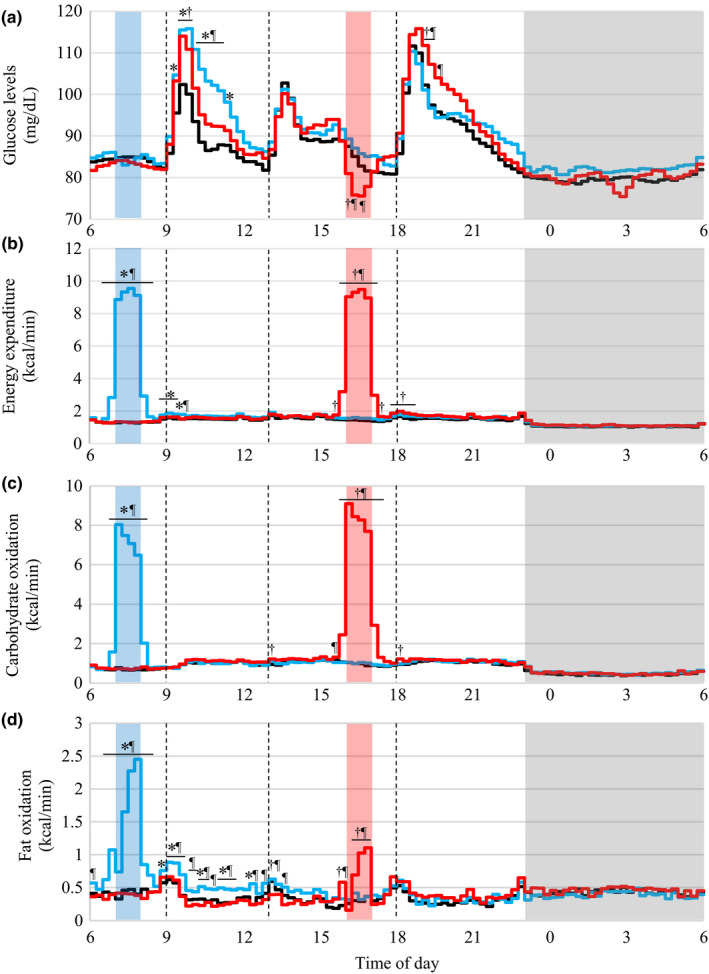
Diurnal variations in glucose levels, energy expenditure, and substrate oxidation. Mean time courses for glucose levels (a), energy expenditure (b), carbohydrate oxidation (c), and fat oxidation (d) are shown (n = 11). Time courses of glucose level, energy expenditure, carbohydrate oxidation, and fat oxidation analyzed using linear mixed‐models ANOVA, the main effects of time (*p* < 0.001), experimental condition (*p* < 0.001), and the time by experimental condition interaction (glucose level; *p* = 0.012, energy expenditure, and carbohydrate and fat oxidation; *p* < 0.001) were significant. Black, blue, and red lines indicate the control, morning exercise, and afternoon exercise trials, respectively. Dashed lines indicate the timing of the meals. Blue, red, and gray areas indicate morning exercise, afternoon exercise, and sleep periods, respectively. *^, †, ¶^ indicate significant difference in the control versus morning exercise trials, the control versus afternoon exercise trials, and the morning versus afternoon exercise trials, respectively

The time course of the glucose levels is shown in Figure 1a. Glucose levels during the morning exercise period did not differ significantly among the 3 trials. After breakfast was consumed, the glucose levels in the 2 exercise trials were higher than that in the control trial from 9:15 (in the morning exercise trial) or from 9:30 (in the afternoon exercise trial) to 10:00 (*p* < 0.05). From 10:00 to 11:15, the glucose level in the morning exercise trial was higher than that in the other 2 trials (*p* < 0.05). During the afternoon exercise period, the glucose levels were lower in the afternoon exercise trial than in the other 2 trials from 16:15 to 16:30 (vs. control trial) or to 16:45 (vs. morning exercise trial, *p* < 0.05). After dinner, the glucose level in the afternoon exercise trial was higher than that in the other 2 trials (from 19:00 to 19:30, vs. control trial; from 19:00 to 19:45, vs. morning exercise trial, *p* < 0.05).

**FIGURE 2:**
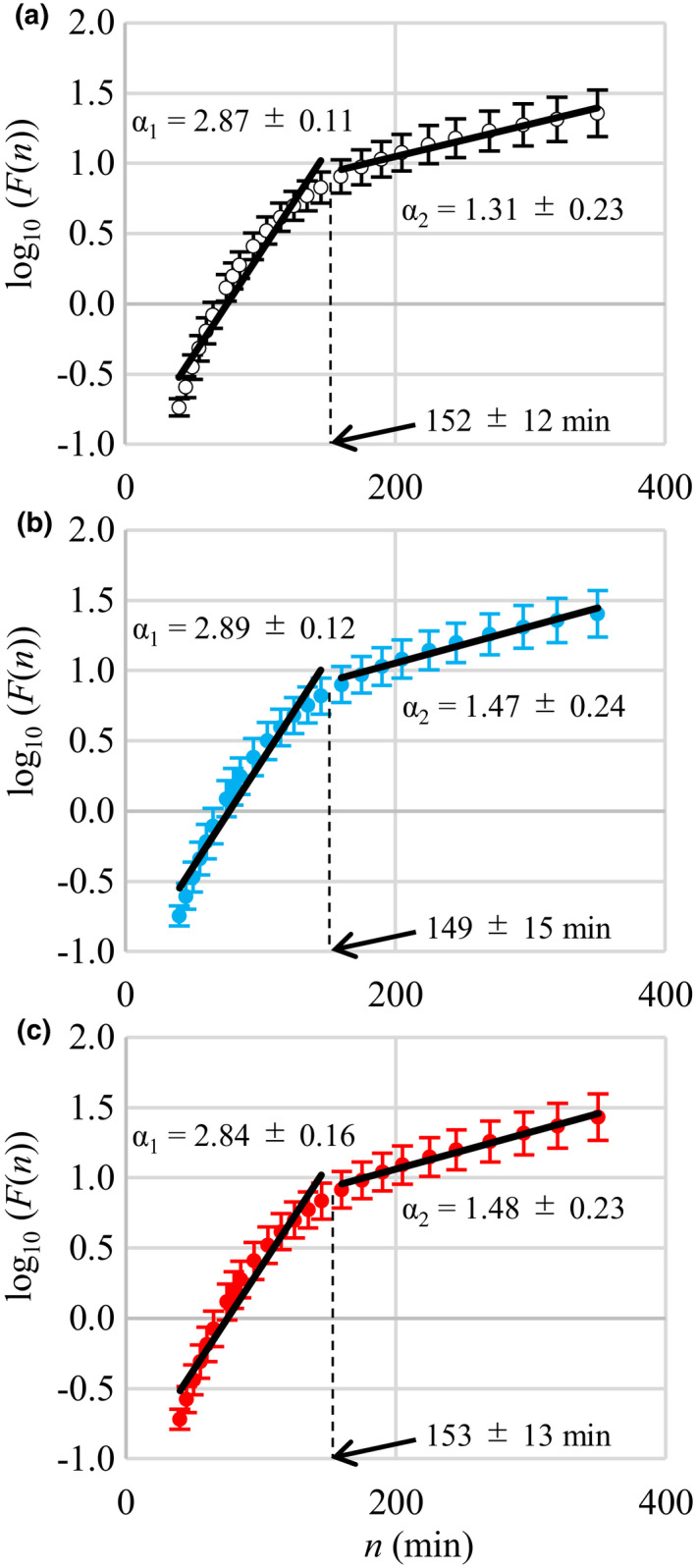
Detrended fluctuation analysis (DFA) plots. The fluctuation functions *F*(*n*) in the control (a), morning exercise (b), and afternoon exercise (c) trials are plotted against the time window (n = 11). α_1_ and α_2_ indicate short‐ and long‐range scaling exponents, respectively. Dashed lines indicate crossover points

Results of indices obtained from the CGM are shown in Table 2. The CONGA2 in the afternoon exercise trial was significantly higher than that in the control trial (*p* = 0.036), but there was no significant difference between the control and morning exercise trials (*p* = 0.115) or the morning and afternoon exercise trials (*p* = 0.394). On the other hand, the 24‐h SD, J‐index, MAGE, CONGA1, and CONGA4 were not significantly different among the 3 trials. The DFA results are shown in Table 2 and Figure 2. The short‐range exponent (α_1_) in the 3 trials was significantly larger than α = 1.5, indicating an uncorrelated reference value (*p* < 0.001), and suggesting that the short‐range glucose fluctuation profile was positively correlated. The α_1_ value was not significantly different among the 3 trials. The long‐range exponent (α_2_) in the 3 trials was significantly larger than α = 1.0 (*p* < 0.05) but not α = 1.5, suggesting that the long‐range glucose fluctuation profile was negatively correlated. The long‐range exponent (α_2_) in the control trial was significantly lower than that in the afternoon exercise trial (*p* = 0.042) and tended to be lower than that in the morning exercise trial (*p* = 0.094), but there was no significant difference between the morning and afternoon exercise trials. The *Fm* and crossover point values were not significantly different among the 3 trials.

**TABLE 2:** Glucose fluctuations and response to exercise and meals

	Control	Morning exercise	Afternoon exercise
Glucose fluctuations			
24 h SD (mg/dl)	8.97 ± 3.84	10.20 ± 3.62	11.77 ± 5.35
J‐index	9.11 ± 1.61	9.98 ± 1.29	10.01 ± 1.83
MAGE (mg/dl)	26.2 ± 11.4	29.9 ± 12.2	32.9 ± 14.2
CONGA1	0.60 ± 0.19	0.66 ± 0.22	0.71 ± 0.25
CONGA2	0.70 ± 0.24	0.83 ± 0.31	0.93 ± 0.35^a^
CONGA4	0.77 ± 0.29	0.84 ± 0.30	0.97 ± 0.40
DFA			
α_1_	2.87 ± 0.11	2.89 ± 0.12	2.84 ± 0.16
α_2_	1.31 ± 0.23	1.47 ± 0.24	1.48 ± 0.23^a^
*Fm*	0.54 ± 0.10	0.54 ± 0.12	0.56 ± 0.16
Crossover point (min)	152 ± 12	149 ± 15	153 ± 13
During exercise period			
Mean glucose			
7:00–8:00 (mg/dl)	85 ± 6	84 ± 7	84 ± 12
16:00–17:00 (mg/dl)	84 ± 9	87 ± 8	78 ± 7^a^, ^b^
Minimum glucose			
7:00–8:00 (mg/dl)	84 ± 2	82 ± 2	82 ± 4
16:00–17:00 (mg/dl)	81 ± 2	84 ± 3	74 ± 2^a^, ^b^
Change of glucose			
7:00–8:00 (mg/dl)	0.3 ± 1.9	−2.6 ± 4.4	−0.8 ± 3.3
16:00–17:00 (mg/dl)	−7.4 ± 3.9	−7.1 ± 3.9	−12.1 ± 4.7^b^
Response to meal			
MIME ∆*G*			
Breakfast (mg/dl)	22 ± 11	37 ± 12^a^	34 ± 15^a^
Lunch (mg/dl)	23 ± 7	18 ± 10	19 ± 6
Dinner (mg/dl)	34 ± 15	29 ± 10	33 ± 9
MIME ∆*T*			
Breakfast (min)	47 ± 26	53 ± 24	42 ± 6
Lunch (min)	45 ± 18	54 ± 35	55 ± 34
Dinner (min)	43 ± 10	55 ± 44	48 ± 13

Main effect of experimental condition compared by 1‐way ANOVA was significant in the CONGA2 (*p* = 0.007), α_2_ (*p* = 0.038), mean (*p* = 0.008), minimum (*p* = 0.008), change in glucose from 16:00 to 17:00 (*p* = 0.002), and MIME∆*G* at breakfast (*p* = 0.001).

^a^ Significant difference versus control trial (*p* < 0.05).

^b^ Significant difference versus morning exercise trial (*p* < 0.05).

From 7:00 to 8:00, the mean, minimum, and change in the glucose level were not significantly different among the 3 trials (Table 2). On the other hand, the mean and minimum glucose levels from 16:00 to 17:00 in the afternoon exercise trial were significantly lower than that in the control (*p* = 0.028 and 0.033, respectively) and morning exercise trials (*p* = 0.034 and 0.038, respectively). The minimum glucose level from 16:00 to 17:00 in the afternoon exercise trial ranged from 63 to 85 mg/dl, and 3 of the 11 subjects experienced hypoglycemia (below 70 mg/dl; Workgroup on Hypoglycemia, American Diabetes Association, 2005). Additionally, the change in the glucose level from 16:00 to 17:00 in the afternoon exercise trial was significantly smaller with a low glucose level than that in the morning exercise trial (*p* = 0.004) and tended to be smaller than that in the control trial (*p* = 0.054). The MIME ∆*G* at breakfast in the control trial was significantly lower than that in the morning (*p* = 0.006) and afternoon exercise trials (*p* = 0.024). The MIME ∆*G* at lunch and dinner and the MIME ∆*T* at all 3 meals were not significantly different among the 3 trials.

### 3.3 Plasma insulin level and correlations between the change in glucose during exercise and pre‐, post‐exercise insulin levels, or pre‐exercise glucose levels

**FIGURE 3:**
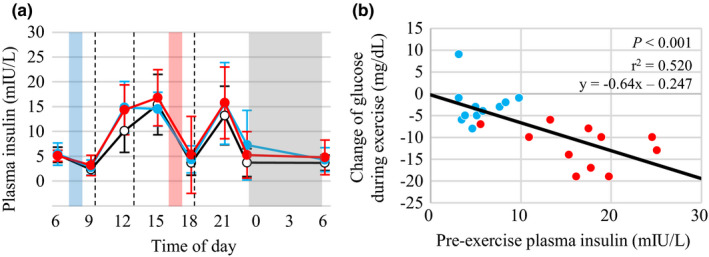
Diurnal variations in plasma insulin levels and relationship between the change in glucose during exercise and the pre‐exercise insulin level. Time course of plasma insulin levels (n = 11) (a). White, blue, and red circles indicate the control, morning exercise, and afternoon exercise trials, respectively. Dashed lines indicate the timing of meals. Blue, red, and gray areas indicate the morning exercise, afternoon exercise, and sleep periods, respectively. The data were analyzed using a linear mixed‐models ANOVA. The main effects of time (*p* < 0.001) and trial (*p* = 0.012) were significant, but the time by experimental condition interaction was not significant (*p* = 0.657). The glucose level change during exercise was plotted against the pre‐exercise plasma insulin levels (b). Blue and red circles indicate individual results in the morning and afternoon exercise trials, respectively

The time course of the plasma insulin levels is shown in Figure 3a. The interaction of time and experimental condition in the plasma insulin levels were not significantly different among the 3 trials. The change in the glucose level during exercise was significantly correlated with the pre‐exercise plasma insulin level (*R* = −0.699, *p* < 0.001; Figure 3b). The change in the glucose level during exercise did not significantly correlate with the pre‐exercise glucose (*p* = 0.341) or post‐exercise insulin (*p* = 0.137) levels.

### 3.4 Energy metabolism

The time courses of energy expenditure, and carbohydrate and fat oxidation over 24 h are shown in Figure 1b–d, respectively. Energy expenditure in the morning exercise trial was significantly higher than that in the other 2 trials (from 6:30 to 8:30; *p* < 0.05 and 9:30 to 9:45; *p* < 0.05) or in the control trial alone (from 8:45 to 9:30; *p* = 0.045). In the afternoon exercise trial, energy expenditure was higher than that in the other 2 trials (from 15:45 to 17:15; *p* < 0.001) or in the control trial alone (from 15:30 to 15:45; *p* = 0.015, 17:15 to 17:30; *p* = 0.008, and 17:45 to 18:45; *p* < 0.05). Carbohydrate oxidation in the morning exercise trial was higher than that in the other 2 trials (from 6:45 to 8:15; *p* < 0.001). In the afternoon exercise trial, carbohydrate oxidation was higher than that in the other 2 trials (from 15:45 to 17:30; *p* < 0.001), the control trial only (from 13:00 to 13:15; *p* = 0.033 and 18:00 to 18:15; *p* = 0.047), and the morning exercise trial (from 15:30 to 15:45; *p* = 0.043). Fat oxidation in the morning exercise trial was higher than that in the other 2 trials during and after exercise until lunch, although the differences at some time points were not statistically significant. Fat oxidation in the afternoon exercise trial was higher from 16:15 to 17:00 than that in the other 2 trials. A significant increase was also observed immediately before the exercise session, probably reflecting activities to prepare for the exercise session.

Results of energy expenditure, substrate oxidation, and energy balance over 24 h are shown in Table 3. Total energy expenditure over 24 h in the 2 exercise trials was higher than that in the control trial (*p* < 0.001). Total carbohydrate oxidation over 24 h in the 2 exercise trials was significantly higher than that in the control trial (*p* < 0.001). Additionally, total carbohydrate oxidation over 24 h in the afternoon exercise trial tended to be higher than that in the morning exercise trial (*p* = 0.074). Total fat oxidation over 24 h in the morning exercise trial was significantly higher than that in the control trial (*p* = 0.003), but there was no significant difference compared with the afternoon exercise trial (*p* = 0.461). Total protein oxidation and energy balance over 24 h were not significantly different among the 3 trials. During the exercise, energy expenditure was not significantly different between the 2 exercise sessions (*p* = 0.72; Table3). On the other hand, carbohydrate and fat oxidation during the exercise were significantly higher in the afternoon (*p* < 0.001) and morning exercise trials (*p* < 0.001) than in the other exercise trials, respectively.

**TABLE 3:** Total energy expenditure and substrate oxidation over 24 h and during exercise session

	Control	Morning exercise	Afternoon exercise
24 h			
Energy intake (kcal)	2029 ± 216	2591 ± 298	2591 ± 298
Energy expenditure (kcal)	1968 ± 189	2638 ± 258^a^	2612 ± 249^a^
Energy balance (kcal)	27 ± 114	−111 ± 173	−85 ± 163
Carbohydrate oxidation (kcal)	1170 ± 152	1649 ± 226^a^	1774 ± 182^a^
Fat oxidation (kcal)	529 ± 136	701 ± 163^a^	557 ± 174
Protein oxidation (kcal)	269 ± 48	288 ± 35	281 ± 32
During exercise session			
Energy expenditure (kcal)		553 ± 78	552 ± 75
Carbohydrate oxidation (kcal)		436 ± 71	502 ± 67^b^
Fat oxidation (kcal)		105 ± 30	38 ± 19^b^

Main effect of experimental condition compared by 1‐way ANOVA was significant for energy expenditure (*p* < 0.001), carbohydrate (*p* < 0.001), fat oxidation (*p* = 0.038), and energy balance (*p* = 0.033) over 24 h.

^a^ Significant difference versus control trial (*p* < 0.05).

^b^ Significant difference versus morning exercise trial (*p* < 0.05).

## 4 DISCUSSION

Indices of glucose fluctuation over 24 h suggested that the effects of exercise on glucose fluctuation depend on when the exercise performed. CONGA2 was significantly higher in the afternoon exercise trial than in the control trial. Among the other fluctuation indices, 24‐h SD, J‐index, MAGE, CONGA1, and CONGA4, the values descended in order from afternoon exercise, morning exercise, and control trials, suggesting larger glucose fluctuation by afternoon exercise, although the differences in each index among the 3 trials were not statistically significant (Table 2). The glucose fluctuation characteristics were also assessed using DFA (Figure 2 and Table 2). The order of the long‐range exponent (α_2_) also descended from afternoon exercise, morning exercise, and control trials and was significantly higher in the afternoon exercise trial than in the control trial. Patients with type II diabetes have a higher α_2_ value (1.65 ± 0.30, Ogata et al., 2006; and 1.747 ± 0.387, Ogata et al., 2012). These results suggest that the negative feedback function is attenuated by exercise, particularly exercise performed in the afternoon, compared with the control trial. Of all the glucose fluctuation indices examined in the present study, CONGA2 and the DFA α_2_ were significantly higher only in the afternoon exercise trial compared with the control trial. These observations in the glucose fluctuation indices may relate to the drop in the glucose level during exercise and decreased meal tolerance after meals following exercise.

Glucose levels during the exercise session were decreased in the afternoon exercise trial, but stable during the morning exercise trial (Figure 1a and Table 2). In previous studies, blood glucose levels decreased during exercise at 60% of $\dot{V}$ O_2_peak for 1 h when the exercise was performed in a fed condition, but were unaffected when the exercise was performed in a fasting condition (Gaudet‐Savard et al., 2007; Poirier et al., 2001). Similarly, 20 sets of 1‐min jogging at lactate threshold (LT) with a 30‐s rest between sets decreased glucose levels during exercise when exercise was performed after breakfast and the subsequent period, but not that before breakfast (Hatamoto et al., 2017). Together, exercise performed after the first meal after overnight fasting and the subsequent period, but not before the first meal, decreases glucose level during exercise. The change in glucose during exercise was negatively correlated with the pre‐exercise plasma insulin levels, suggesting a role of insulin to induce hypoglycemia during exercise (Figure 3b). Glucose intake 30–60 min before the start of an exercise increases insulin concentrations and decreases blood glucose levels during exercise (Costill et al., 1977; Foster et al., 1979). Carbohydrate oxidation during the afternoon exercise session was also higher than that during the morning exercise session (Table 3), which is consistent with previous studies assessing the effect of exercise performed in either before breakfast or after breakfast and the subsequent period on energy metabolism (Iwayama et al., 2017; Iwayama, Kawabuchi, et al., 2015; Iwayama, Kurihara, et al., 2015; Shimada et al., 2013). Therefore, exercise performed in the afternoon with high insulin levels increased glucose oxidation and reduced glucose levels during exercise, which may contribute to the increase in CONGA2 and α_2_ in DFA.

Glucose levels following the postprandial peak after breakfast and dinner were higher when exercise was performed before the meal (Figure 1a). On the other hand, after breakfast, carbohydrate oxidation was not significantly different among the 3 trials. After dinner, carbohydrate oxidation in the afternoon exercise trial was higher than that in the control trial and was not significantly different compared with the morning exercise trial (Figure 1c). In other words, suppressed meal tolerance due to pre‐meal exercise was not accompanied by reduced carbohydrate oxidation. A previous study reported that 45 min of exercise (3 bouts of 15 min treadmill or cycling exercise at 75% of $\dot{V}$ O_2_peak and 5 min rest) in a fasting condition increases the glucose AUC over 3 h during an oral glucose tolerance test (OGTT) 30 min after the exercise in middle‐aged subjects with normal glucose tolerance (King et al., 1995). In another study in endurance‐trained healthy young men, exercise was performed in a fasting condition at 70% of $\dot{V}$ O_2_peak for 60 min, followed by OGTT 30 min after the exercise (Rose et al., 2001). The exercise increased the glucose AUC over 2 h and was associated with increased glucose appearance. In that study, the appearance rate of exogenous glucose during the OGTT was measured using a double‐tracer technique. Whereas in the previous studies (King et al., 1995; Rose et al., 2001) the decrease in glucose tolerance was induced by performing the exercise after overnight fasting, in the present study the decrease in meal tolerance was induced by performing the exercise in both morning and afternoon. Therefore, exercise performed before a meal may decrease meal tolerance, at least in part, through increased exogenous glucose appearance, regardless of the nutritional status.

Another factor that affects the postprandial glucose level is meal size. To achieve an individual energy balance over 24 h during indirect calorimetry, the meal size in the exercise trials was larger than that in the control trial. Therefore, glucose levels from 9:30 to 10:00 and MIME*∆G*, an increase of postprandial glucose, after breakfast was higher in the exercise trials than in the control trial (Figure 1a and Table 2). Glucose levels from meal intake to the postprandial peak and MIME*∆G* after lunch and dinner, however, did not differ significantly between the control and exercise trials. The increase in glucose levels after the second and subsequent meals during the day was affected by the previous meal, called the second‐meal phenomenon (Franz, 2001). Therefore, the increase in the glucose levels observed in the present study was most sensitive to meal size at breakfast and may be masked by the second‐meal phenomenon at lunch and dinner. On the other hand, the difference in glycemic homeostasis can be compared between the 2 exercise trials in which the same meal was consumed. Postprandial glucose excursion was significantly greater in the trial with the exercise performed prior to the meal compared with the other exercise trial, from 60 to 135 min after breakfast and from 60 to 105 min after dinner (Figure 1a). Therefore, the decrease in the meal tolerance following exercise in the present study may have been induced by exercise rather than meal size.

Several previous studies assessing the effects of exercise on glucose fluctuation examined only a few indices. In patients with insulin‐treated type 2 diabetes, CONGA1, CONGA2, and CONGA4 were not affected by combining resistance and interval aerobic exercise performed at 12:00 (Praet et al., 2006). Additionally, the effects of exercise on MAGE differ among studies. Treadmill exercise at a target intensity of 40% of the heart rate reserve for 20 min performed 30 min after dinner (Li et al., 2018) and that at 5.0 km/h and a 0.5% incline for 50 min performed 3 to 5 h after lunch (Rees et al., 2019) does not affect MAGE. On the other hand, MAGE is decreased by both high‐intensity interval (repetitions of 3 min at 40% of $\dot{V}$ O_2_peak followed by 1 min at 100% of $\dot{V}$ O_2_peak, that is, total of 15 high‐intensity bouts, mean calculated relative intensity 55% of $\dot{V}$ O_2_peak) and moderate‐intensity continuous (55% of $\dot{V}$ O_2_peak) treadmill exercise for 60 min performed 120 min before breakfast compared with no‐exercise control (Terada et al., 2016). The same exercises performed 80 min after breakfast, however, do not affect MAGE. In addition, MAGE is decreased by 2 sets of 6 resistance exercises (at 80% of 1‐repetition maximum) and combined exercise (20 min each of aerobic exercise at 80% of heart rate reserve and 1 set of 6 resistance exercises) for 40 min performed in a fasting condition, but is not affected by aerobic exercise for 40 min (Minnock et al., 2020). Although the results of the previous studies were inconsistent, if the exercise had any effect on the glucose fluctuation, the glucose fluctuation indices were decreased. The present study, however, demonstrated that exercise increased the glucose fluctuation indices compared with the control trial. Of note, in the present study, the meal size was increased in the exercise trials to achieve an individual energy balance over 24 h, while in the previous studies, the same meals were provided during trials with or without an exercise session (Li et al., 2018; Minnock et al., 2020; Praet et al., 2006; Rees et al., 2019; Terada et al., 2016). It is possible that the difference in the energy balance or meal size plays a role in controlling glucose fluctuation.

The present study has some limitations. First, the indwelling catheter used for blood sampling may have increased levels of interleukin‐6, which is an inflammatory biomarker (Chabot et al., 2018), and thus, the results of the present study might be influenced by inflammation. Second, the subjects in the present study were young healthy men without insulin resistance, and the effects of exercise might not be clinically meaningful. Third, difference of the effect of exercise timing on glucose fluctuation could be attributed to the nutritional status and/or diurnal rhythm in glucose homeostasis.

## 5 CONCLUSIONS

Based on the multiple glucose fluctuation indices, the present study demonstrated that the effects of exercise on glucose fluctuation depend on the timing of the exercise. Glucose levels were slightly less stable when the exercise was performed in the afternoon, with higher insulin levels than morning (before breakfast). The different effects of the timing of exercise on glucose fluctuations may be due to differences in the glucose level changes during exercise. We also revealed that exercise attenuates meal tolerance after the next meal, independent of the nutritional status during the exercise, and substrate oxidation during the meal. The results of the present study may provide beneficial information for those who need to maintain glycemic homeostasis, such as patients with diabetes and cardiovascular disease. However, the optimal timing of exercise for maintaining glycemic homeostasis, whether repeated bouts of exercise have the potential to improve symptoms related to glycemic homeostasis, and clinical significance of exercise timing in the population with insulin resistance remain to be determined.

## DISCLOSURE

No conflicts of interest, financial or otherwise, are declared by the authors.

## AUTHOR CONTRIBUTIONS

Y.T., H. Ogata, Y.N., M.S., and K. Tokuyama conceived and designed the research; Y.T., H. Ogata, I.P., A. Ando., M.K., K.Y., C.S., A. Araki., H. Osumi, S.Z., and K. Takahashi performed the experiments; Y.T., H. Ogata, J.S., and K.Y. analyzed the data; Y.T., H. Ogata, and K. Tokuyama; Y.T. prepared the figures; Y.T., H. Ogata, A.I., and K. Tokuyama drafted the manuscript; Y.T., A.I., and K. Tokuyama edited and revised the manuscript; Y.T. and K. Tokuyama approved the final version of manuscript.
